# Can We Validate the Results of Twin Studies? A Census-Based Study on the Heritability of Educational Achievement

**DOI:** 10.3389/fgene.2017.00160

**Published:** 2017-10-26

**Authors:** Inga Schwabe, Luc Janss, Stéphanie M. van den Berg

**Affiliations:** ^1^Department of Research Methodology, Measurement and Data Analysis (OMD), University of Twente, Enschede, Netherlands; ^2^Department of Methodology and Statistics, Tilburg University, Tilburg, Netherlands; ^3^Center for Quantitative Genetics and Genomics, Department of Molecular Biology and Genetics, Aarhus University, Aarhus, Denmark

**Keywords:** census-based heritability, educational achievement, behavior genetics, twin studies, pedigree based mixed models

## Abstract

As for most phenotypes, the amount of variance in educational achievement explained by SNPs is lower than the amount of additive genetic variance estimated in twin studies. Twin-based estimates may however be biased because of self-selection and differences in cognitive ability between twins and the rest of the population. Here we compare twin registry based estimates with a census-based heritability estimate, sampling from the same Dutch birth cohort population and using the same standardized measure for educational achievement. Including important covariates (i.e., sex, migration status, school denomination, SES, and group size), we analyzed 893,127 scores from primary school children from the years 2008–2014. For genetic inference, we used pedigree information to construct an additive genetic relationship matrix. Corrected for the covariates, this resulted in an estimate of 85%, which is even higher than based on twin studies using the same cohort and same measure. We therefore conclude that the genetic variance not tagged by SNPs is not an artifact of the twin method itself.

## Introduction

There are evident individual differences in children's educational achievement. While some learn at such a fast pace that they are bored at school, others are struggling in each subject. This inevitably leads to the nature vs. nurture debate: To what extent can we explain these differences in school performance by genetic and environmental influences? One of the most-often used methods to quantify the relative influence of nature and nurture is the twin study design. Twin pairs are either identical (monozygotic, MZ) and share the same genetic material, or non-identical (dizygotic, DZ) and share on average only half of the segregating genes. When identical twins are more similar (e.g., in school performance) than non-identical twins, this implies that genetic influences are at play. We then conclude that to some extent, individuals are different from each other at least in part because of differences in genotypes. Twin researchers often use the so-called ACE model that decomposes variance in observed measures into additive genetic variance (A), common-environmental variance (C) and unique-environmental variance (E). Common-environmental variance represents variance due to the environmental influences that make siblings similar but cannot be attributed to their genetic resemblance. Unique-environmental variance refers to variance explained by non-genetic influences that render siblings different. Heritability is then defined as the proportion of the total observed variance that can be explained by genetic differences. Twin studies are usually based on large twin registries where study participation is voluntary. Results of twin studies suggest that educational achievement is a highly heritable trait. Including 61 studies from 11 cohorts and 7 continents/countries (USA, Scandinavia, Australia, UK, China and the Netherlands), de Zeeuw et al. (2015) conducted a large meta-analysis on twin studies of educational achievement in primary school. Based on up to 5,330 MZ and 7,084 DZ twin pairs, the heritability in general educational achievement was estimated at 66%. Common-environmental effects explained only 12% of the variation in general educational achievement and unique-environmental influences 22%. Heritability estimates however differed between countries. For example, while heritability was high in all educational domains in the Netherlands, this was not true for the USA and the UK. As the authors note, this might be due to differences in educational opportunities: While in the USA and the UK, private public schools have the right to select their students and charge tuition, in the Netherlands all public as well as private schools have to comply to the same standards. This relatively homogenous education environment may restrict variation in school environments and therefore lead to a higher heritability in the Netherlands compared to the USA or the UK (see also de Zeeuw et al., 2015).

Over the past decade, molecular genetic studies that tried to identify the genetic variants responsible for the high heritability of educational achievement have revealed that there are no genetic variants with large effects. As, usually, multiple genes are involved in the inheritance of a complex trait (a phenomenon also termed *polygenic* inheritance), studies often use *polygenic scores* rather than assessing the pure presence or absence of a single gene mutation. A polygenic score aggregates the effect of all significant single nucleotide polymorphisms (SNPs) into one genotypic score for a particular trait (Dudbridge, 2013; de Vlaming and Groenen, 2015). The polygenic score is calculated for every participant in the sample separately, using regression weights resulting from earlier genome-wide association studies (GWAs) that identified genetic variants that have a significant relationship with the trait of interest.

One of the largest (*N* = 126,559) GWAs on educational achievement was performed on *years of education*, a proxy measure for the trait. They found that the DNA variant with the largest effect size accounted for 1% of the variance in years of education, but the polygenic score accounted for only 2% in a replication sample (Rietveld et al., 2013). In a follow-up study (*N* = 329,000), a revised polygenic score based on a new GWAS was used, which almost doubled the effect size: 3.9% of the variance in years of education could be explained in an independent sample (Okbay et al., 2016). In a Dutch sample of about 1,000 children tested at age 12, the polygenic score calculated based on the GWAs of Rietveld et al. (2013) accounted for about 2% of the variance in educational achievement. The latest genetic study used the effect size estimates from Okbay et al. (2016) to calculate a polygenic score for each individual in a sample of 5,825 unrelated UK students with known years of education as well as educational achievement scores on assessments of the national curriculum at ages 7, 12, and 16 (Selzam et al., 2017). Similar to the results by Okbay et al. (2016) they found that the polygenic score accounted for only approximately 4% of the variance of years of education in independent samples. The polygenic score however explained more variance in educational achievement test scores, increasing from age 7 (3%) to age 12 (5%) and to age 16 (9%). Although the use of more modern techniques such as estimating variance explained by all common SNPs showed a significant proportion of explained variance in educational achievement (i.e., 27% for reading and 52% for mathematics, see Davis et al., 2015), there is still a gap between the variance in educational achievement explained by all SNPs taken together and the heritability as observed in twin studies (see also de Zeeuw et al., 2015). Thus, one major question is whether perhaps the twin method leads to inflated heritability estimates. So what problems are there with the twin method, and what alternative approaches do we have to validate or falsify the heritability estimates based on the twin method?

Twins tend to have a lower birth weight than singletons (see e.g., Alexander et al., 1998). de Zeeuw et al. (2012) found that Dutch twins with lower birth weight and small for gestational age performed more poorly, among others, on a standardized measure for educational achievement (the *Eindtoets Basisonderwijs* test) than Dutch twins with a normal birth weight. Furthermore, singletons received higher rating from their teachers for, among others, arithmetic skills. These differences could however be largely accounted by the birth order within a family: Twins who were first in birth order had the same (or even higher) ratings as their non-twin sibling. Contrary, twins who were not first in birth order had lower rating than their non-twin sibling(s). Second, participation in registries is *voluntary* and might therefore introduce self-selection bias (see e.g., Joseph, 2014). This is indeed the case. For instance, compared to the general population, parents of twins that register have a relatively high average socio-economic status (SES) (Joseph, 2014). Since SES has consistently been associated with higher academic achievement throughout childhood and adolescence (see e.g., White, 1982; Sirin, 2005), such selection bias results in lower variance and potentially to under- or overestimated heritability. Third, Gielen et al. (2008) showed that, compared to a reference data set of all Dutch live-born twins, twins who are registered with the Netherlands Twin Register (NTR) have a somewhat higher mean birth weight and gestational age. It is important to note, however, that in general results are mixed. While aforementioned Dutch studies show that twins registered with twin registries are not a completely representative sample of all twins and twins are not a representative sample of the entire population, other studies (e.g., Christensen et al., 2006) show no differences between twins and singletons, for other populations and other phenotypes.

One way to validate the twin method is to compare results with those of an adoption design: comparing educational achievement in children with that of their foster parents and, if possible, with that of their biological parents. However, for childhood phenotypes like school test scores, it is hard to find standardized measures that can be compared meaningfully across two generations: tests change over time and test scores get lost.

What we need is a population sample (1) that shows no self-selection bias, (2) of children that are more representative regarding suboptimal pre- and perinatal circumstances, (3) with which we can draw inference regarding the influence of genetics, and (4) where test scores are available and can be compared meaningfully. Here we investigated whether earlier Dutch twin study results on educational achievement can be validated and generalized to the general Dutch population by making use of a sample of *all* Dutch children that within a certain time-frame of seven years received a well-known standardized educational achievement test in the Netherlands. The heritability was estimated using the known family relationships between all of these children and applying linear mixed models (Thompson, 2008). The test scores were collected by schools and made available by the *Netherlands Bureau of Statistics* that also provided information on family relationships and covariates. In the discussion, we will compare these results with the results on the *same* standardized achievement test that are based on data from twins registered at the NTR from the *same* birth cohort.

## Method

### Phenotypic data

The phenotypic data consisted of achievement scores from all pupils registered in the Dutch primary education system during the years 2008 through 2014 using the *Eindtoets Basisonderwijs* test, which is an achievement tests that is administered yearly in the final year of primary school (around age 12). The *Eindtoets Basisonderwijs* consists of 290 multiple choice items in four different subjects [language, arithmetic/mathematics, study skills and world orientation (optional)] and assesses what a child has learned during primary school. Together, the performance scales result in a standardized total score between 500 and 550 that is used to give an advice on the choice for secondary education. From these scores we selected only the scores between 500 and 550, since other values cannot be valid. Thus, we obtained a total of 902,884 test scores with 41,242 test scores from 2008, 93,642 from 2009, 146,363 from 2010, 152,971 from 2011, 156,356 from 2012, 154,821 from 2013, and 157,489 from 2014. For some children, two scores were found in different years (*N* = 1,204). In those cases, the earliest observed score was selected and used for further analysis, resulting in a total of 902,884–1,204 = 901,680 scores. For every pupil in our dataset, the sex, migrant background and adoption status were determined, using information obtained from the Netherlands Bureau of Statistics. Parentage information from the Netherlands Bureau of Statistics (i.e., what was known at the local council) was available for 899,437 pupils, but children that were known to be adopted were left out of the analysis, reducing the number of phenotyped pupils to a total of 894,750. On the basis of the census data, it was not possible to determine zygosity (i.e., MZ or DZ) of twins included in the dataset such that there are some pairs of individuals in our analysis that were assumed fraternal while they were actually identical.

### Pedigree data

Pedigree data (i.e., for each individual the ID of the father and/or mother) was available for 14,875,512 individuals. Individuals that were known to be adopted themselves were given unknown parents (*N* = 16,334). The pedigree was simplified by omitting individuals that were involved in a loop (*N* = 593): for instance persons being their own grandparent. The pedigree was further reduced by omitting branches without phenotypic information (non-informative branches: nonphenotyped individuals with no phenotyped children and with no phenotyped grandchildren). In this way, all relations in the phenotypic data that were either full siblings, half siblings, half cousins, or full cousins were identified, but all other relations were assumed to be nonexistent (i.e., assuming a genetic correlation of 0). Data on great-grandparents of phenotyped children was available but deemed too unreliable by the Netherlands Bureau of Statistics and therefore ignored (i.e., great-grandparents of phenotyped children were assumed unknown).

### Covariates

As covariates, we used *Group size* (i.e., number of pupils that a child shared a classroom with), *School denomination* (i.e., type of school), *Migrant status* (i.e., first, second or third generation), *Sex* and *SES* for which we used a variable that indicated the weight that is, depending on the educational level of the parents, given to a pupil by the Dutch government as a proxy measure. In the following, these covariates are described in more detail.

Information on all covariates was retrieved from the Netherlands Bureau of Statistics. All phenotyped children had complete data on covariates, except that children from special education (primary education in smaller groups for children with learning or behavioral problems, *N* = 1,623) did not have a covariate value for the weight covariate (i.e., the SES proxy used here, see the following for more detail). These children were excluded from the analysis with covariates, which led to a total sample size of 893,127 children.

The covariate *Group size* measured the number of pupils that the pupil shares a classroom with most of her or his time during primary education. The covariate *School denomination* distinguishes between the different types of schools. Overall, the Dutch education can be divided into public schools, private schools and special schools (publicly funded). Public schools provide secular education, but can also teach around specific pedagogical principles (e.g., Montessori). Private schools are inspired by a religion (e.g., Protestant-Christian, Reformed or Roman-Catholic). Special schools are schools that are distinct from both public and private education. They are generally administered by an independent board, as opposed to government authority, while still receiving government funding. At the Netherlands Bureau of Statistics, these three types are further divided into 37 subcategories. In our data, 18 different school denominations occurred. Following the same coding as at the Netherlands Bureau for Statistics, in our data, the four largest school denominations in our data were the following: ABZ (4.49%), OPB (30.20%), PC (23.83%), and RK (36.35%). The category ABZ captures general special schools (i.e., *Algemeen bijzonder*), which is neutral education in the sense that education is not bound to ideologies or social movements. OPB (i.e., *Openbaar*) refers to public schools, PC are Protestant-Christian schools (i.e., *Protestants-Christelijks*) and RK are Roman-Catholic oriented schools (i.e., *Rooms-Katholiek*). For a complete list of all denominations that occurred in the data, the reader is referred to the official documentation at the website of the Netherlands Bureau of Statistics. The three-level covariate *Migrant status* indicates whether a pupil is either a first generation migrant (1), a second generation migrant (2), or neither (0). A first generation migrant was defined as a pupil born abroad with at least one parent born abroad. A second generation migrant was defined as a pupil born in the Netherlands with at least one parent born abroad. All other pupils got a score of 0. This variable was treated as a categorical variable with the 0 category serving as the reference category, as was the *Sex* covariate (i.e., Female or Male) with Female serving as reference category.

In the Dutch educational system, depending on the educational level of the parents, pupils are assigned to one of the following weights: 0.25, 0.30, 0.40, 0.70, 0.90, and 1.20. A weight of 0.25 is given to pupils with poorly educated indigenous parents who both have a schooling to our through pre-vocational exam. Pupils whose parents did not exceed the level of practical training or pre-vocational education from the basic vocational program or the management vocational program get a weight of 0.30. A weight of 0.40 is assigned to pupils who are staying in a boarding school or foster home and whose father or mother is or has been engaged in a skipper company. When the parents work in the circus or fair business and live or have lived in a caravan, a weight of 0.70 is assigned and pupils with poorly educated immigrant parents with an education up to graduation from preparatory vocational education get a weight of 0.90. A weight of 1.20 is assigned to pupils with one parent that only finished primary school and whose other parent finished only the highest pre-vocational education of the basic vocational program or the management vocational program. All other children get a weight of 0.00. This weight variable was used as a proxy for the *SES* of the pupils.

Table 1 gives an overview of all categorical environmental covariates that were used in this paper. For a more detailed description of the covariates used, the reader is referred to the official documentation at the website of the Netherlands Bureau of Statistics.

**Table 1:** Overview of all categorical environmental covariates.

	**N (% of total sample)**
**SEX**	
Female	447,614 (50)
Male	445,513 (50)
**SES**	
Weight of 0.00	775,601 (86.84)
Weight of 0.25	3,648 (0.41)
Weight of 0.30	63,860 (7.15)
Weight of 0.40	61 (0.01)
Weight of 0.70	61 (0.01)
Weight of 0.90	4,496 (0.50)
Weight of 1.20	45,400 (5.08)
**MIGRANT STATUS**	
0	697,656 (78.11)
1	20,620 (2.31)
2	17,4851 (19.58)
**DENOMINATION OF SCHOOL**	
ABZ	40,063 (4.49)
ASF	1,024 (0.11)
EVA	407 (0.05)
EVB	204 (0.02)
GEV	8,638 (0.97)
HIN	1,239 (0.14)
IC	232 (0.03)
ISL	5,140 (0.58)
JOO	209 (0.02)
OPB	269,696 (30.20)
PC	212,870 (23.83)
REF	20,057 (2.25)
RK	324,690 (36.35)
SCA	121 (0.01)
SOC	41 (< 0.01)
SOP	176 (0.02)
SOR	501 (0.06)
SPR	7,819 (0.88)

*Overview of covariates. N, total number of students; ABZ, general special schools; OPB, public schools; PC, Protestant-Christian schools; RK, Roman-Catholic oriented schools (for a complete list, see the official documentation at the website of the Netherlands Bureau of Statistics)*.

### Genetic model

For the analysis of the data, pedigree-based mixed models were used, a statistical method that is borrowed from the field of animal genetics (Thompson, 2008). In a pedigree-based mixed model, the proportion of phenotypic variance, $\sigma_{P}^{2}$, that is due to additive genetic differences among individuals ($\sigma_{A}^{2}$) and the proportion that is interpreted as arising from environmental effects ($\sigma_{E}^{2}$) is estimated. The narrow-sense heritability, *h*^2^, is then defined as the proportion of phenotypic variance that can be explained by additive genetic (i.e., $\frac{\sigma_{A}^{2}}{\sigma_{P}^{2}}$). Next to the trait scores, the only information that is necessary to use pedigree-based mixed models comes from the expected genetic relationships between all individuals. Based on these expected genetic relationships, a genetic relationship matrix is constructed. For more detailed treatments of pedigree mixed models, the interested reader is referred to Lynch and Walsh (1998) or Kruuk (2014). Note that this model is statistically equivalent to the ACE model for twins, except that the common environmental component C is assumed to be zero: fitting an AE model to twin data would yield the exact same result as applying this linear mixed model to twin data.

### Statistical analysis

For this article, we used a Bayesian parametrization of the above described model. In Bayesian analysis, statistical inference is based on the joint posterior density of the model parameters, which is proportional to the product of the likelihood function and a prior probability distribution (for an introduction to Bayesian statistics see e.g., Bolstad, 2007 or Box and Tiao, 1992). A prior probability distribution represents information about an uncertain parameter before any data have been observed. In this particular application, uninformative prior distributions were chosen, meaning that they expressed no information about the parameters of our model. Therefore, posterior point estimates presented here are close to maximum likelihood estimates.

For each parameter, the mean and standard deviation of the marginal posterior distribution was calculated.

Additional to the full model which included all covariates, a simple model that contained only variance components and an intercept, was fitted. In this analysis, all available data was used (*N* = 894,750). For the data preparation, the software package R (R development core team, 2008) and in particular the R packages pedigree (Coster, 2013) and kinship2 (Therneau and Sinnwell, 2015) were used. The MCMC estimation was done using the Bayz software (Janss, 2011), which is freely available at http://www.bayz.biz/. We used 10,000 burn-in iterations, and then 50,000 iterations for inference, saving every 100th iteration. This was chosen on the basis of a large number of trial runs with various starting points to check for good MCMC sampling behavior.

## Results

The posterior means for the variance components, $\sigma_{A}^{2}$ and $\sigma_{E}^{2}$, as well as estimated heritability (i.e., *h*^2^, defined as $\frac{\sigma_{A}^{2}}{\sigma_{P}^{2}}$, where $\sigma_{P}^{2}\;=\;\sigma_{A}^{2}+\;\sigma_{E}^{2}$) for both the full model (with covariates) and the empty model (without covariates) are displayed in Table 2. Regression coefficients for the full model can be found in Table 3. The intercept, μ, was estimated at 535.03 with a standard deviation of 0.01 in the empty model.

**Table 2:** Posterior means (and SDs) for variance components and heritability for empty and full model.

	**$\sigma_{A}^{2}$**	**$\sigma_{E}^{2}$**	***h*^2^**
**EMPTY MODEL**			
Posterior mean (SD)	89.10 (0.41)	5.59 (0.33)	0.94 (0.004)
**FULL MODEL**			
Posterior mean (SD)	75.83 (0.36)	13.21 (0.29)	0.85 (0.003)

*Eindtoets basisonderwijs testscores: Posterior means (SD) of variance components and heritability for the empty model (excluding environmental covariates) and the full model (including environmental covariates)*.

**Table 3:** Regression coefficients for the full model.

	**Posterior mean**	**Posterior SD**
Intercept	537.24	0.05
Sex	0.35	0.02
Weight	−6.26	0.04
Group size	0.03	0.001
Migrant status = 0	0	-
Migrant status = 1	−1.97	0.07
Migrant status = 2	−1.50	0.03
ABZ	0	-
ASF	−1.88	0.31
EVA	−3.22	0.45
EVB	−3.84	0.73
GEV	−1.82	0.13
HIN	−0.47	0.28
IC	−4.88	0.65
ISL	−0.76	0.16
JOO	0.01	0.75
OPB	−2.07	0.05
PC	−1.93	0.05
REF	−1.81	0.09
RK	−1.58	0.05
SCA	−4.61	0.96
SOC	−1.35	1.69
SOP	−3.44	0.73
SOR	−1.12	0.44
SPR	−2.65	0.12

*Eindtoets basisonderwijs testscores: Posterior means and standard deviations (SD) of fixed effects for the full model. ABZ, general special schools; OPB, public schools; PC, Protestant-Christian schools; RK, Roman-Catholic oriented schools (for a complete list, see the official documentation at the website of the Netherlands Bureau of Statistics). For the covariates Sex, Migration status and School denomination, dummy variables were used. The reference category for Sex is Female*.

The results for the empty model without any covariates suggested that the largest part of the variance could be explained by genetic differences, resulting in a posterior mean for *h*^2^ of 0.94 in the empty model. Of the total variance, 89.1 + 5.6 = 94.7, around 6% (94.7–75.8–13.2 = 5.7) could be explained by the covariates.

When these covariate effects were taken into account, the heritability estimate decreased to a posterior mean of 0.85 for the full model, suggesting that part of the variance shared by family members that is captured in the $\sigma_{A}^{2}$ component in the empty model can be explained by environmental covariates. The results of the full model furthermore showed that SES had a clear impact on individual differences in test scores: Pupils with the largest weight value (1.20, i.e., a low SES) scored on average more than six points lower than their fellow pupils with a weight of 0. Furthermore, students from *JOO* schools (i.e., with a Jewish denomination), *ABZ* schools, *HIND* schools (i.e., Hindu) and *ISL* (i.e., Islamite) showed the highest average scores, whereas the lowest averages were observed in *IC* (i.e., interconfessional), *SCA* (i.e., protestant/catholic), *EVB* and *EVA* (i.e., evangelical) and *SOP* (i.e., public/protestant) schools. Note that these effects are corrected for immigrant status and SES. First and second generation immigrant pupils clearly showed scores of around 1.5–2.0 points lower than non-immigrant pupils. Group size was clearly but weakly related to performance: for every extra pupil in the classroom, the expected test score increased by 0.03 points. Note that in this analysis of the full model, special primary education pupils were left out. Boys scored on average 0.35 points higher than girls. In sum: the majority of the variance in test scores was explained by family relationships, and only a small part (around 6%) by the covariates. After correction for the covariate effects, 85% of the remaining variance could be explained by genetic differences.

## Discussion

In the Netherlands, the results of a national educational achievement test, the *Eindtoets basisonderwijs*, partly determine the level of secondary education suitable for a child. Several twin studies have looked at the heritability of individual indifferences on this test, but due to self-selection bias and possible differences in singletons and twins, these results might not generalize to the general population of Dutch pupils. Here we determined the heritability of test scores using population-wide census data. For the estimation, pedigree-based mixed models were used, a method borrowed from the field of animal genetics. We found a heritability of 0.94. When corrected for several school-related covariates, this estimate dropped to 0.85. How does this fairly high heritability estimate compare to that based on twin studies?

A few studies have been done on the same phenotype in the same birth cohort of Dutch children. For example, Bartels et al. (2002) conducted a twin study of 1,495 Dutch twins from the NTR from the birth cohorts 1998–2001 on the sum scores of the same test investigated here at age 12 (*Eindtoets basisonderwijs*). They found that genetic influences explained 57% of the variance in test scores and environmental influences 43%. Twenty-seven percent of the environmental variance could be explained by common-environmental influences and 16% by unique-environmental influences. Schwabe et al. (2016) analyzed the sum scores of 990 Dutch twin pairs from a similar birth cohort (1997–2000) from the NTR but also investigated the effect of the sex of a twin and specific covariates (i.e., school denomination, pedagogical philosophy, school size). Similar to the findings of Bartels et al. (2002), the results suggested that differences in test scores can be explained mainly by genetic influences (66%). Interestingly, while the heritability estimate dropped from 0.94 to 0.85 in the census-based analysis, including covariates did not change the heritability estimate in the Schwabe et al. (2016) study. This might be explained by the lower statistical power of the Schwabe et al. (2016) study, leading also to a lower variance of the covariate distribution: For example, 74% of the twins followed regular education and the school's denomination was Roman-Catholic for 31% of the twins.

Overall, the results of twin studies imply that individual differences in the scores on the *Eindtoets Basisonderwijs* test can be largely explained by genetic differences: Estimated heritability ranges from 60% (Bartels et al., 2002) up to 74% (de Zeeuw et al., 2016). Earlier research furthermore suggests that the finding of a high heritability can be generalized not only to the total score of the *Eindtoets Basisonderwijs*, but also to its subscales (see e.g., de Zeeuw et al., 2016; Schwabe et al., 2017). When we compare these heritability estimates to the estimate of 85% in this study, we can conclude that the high estimates resulting from the twin method are not simply an artifact of self-selection or because of any important difference between twins and singletons. Twin-based heritability estimates are not inflated, since an estimate based on a sample from the entire population (including twins and singletons) is even higher.

However, one might rightly ask whether our analysis of this sample does not have other problems; in other words, that our estimate might also be inflated. There are several shortcomings that should be kept in mind when interpreting the estimate of 85%. First, our analysis is based on parentage information based on census data: mothers and fathers are not asked whether they are the biological parents. We simply use the information that is known at the local council. Although we excluded adopted children and parents from our analyses, there might still be a significant number of parents that are not the biological parents. Of course this bias is there also with twin registers but less severe since parents there are explicitly asked whether they are (or think they are) the biological parents. But if there are non-biological relationships in our analyses, this can only have led to an underestimation of heritability.

Second, since our analysis included all pupils with available data, there are a number of monozygotic twin pairs (or multiples) in our data. As on the basis of census data, zygosity cannot be determined, there are some pairs of individuals in our analysis that were assumed fraternal while they were actually identical. This may have led to overestimation of the heritability. The seriousness of this bias is however proportional to the number of MZ twins, which is only a small part of the Dutch population (roughly one in every 200 births).

Third, as we have used only biological parentage data in our analysis (parentage on paper) we have not taken into account whether sibling pairs and half-sibling pairs actually share the same home environment, nor whether cousins share the same household (could be true for some families), nor whether nonrelated children share the same household. We were therefore not able to estimate non-genetic variance that is shared due to being brought up in the same home environment. If ignored, this variance, as far as it is not related to the covariates that we used in the analysis, ended up in the heritability estimate. This may have led to an overestimation of heritability. Note however, that the shared environmental component was rather small (8 and 16%, respectively) in both the de Zeeuw et al. (2016) and the Schwabe et al. (2016) studies that looked at the same birth cohort. Applying this to the results of this article, this would decrease heritability from 94% (that includes shared environmental variance) to around 77–85%, which would mean that the result is not at odds with the results found by earlier twin studies.

Put differently, if we acknowledge that we can only estimate A + C together in our analysis (that is, in the empty model), and when we compare that to the A + C estimates based on the twin studies using the exact same birth cohorts, the results are rather similar: 94% here, 74 + 8 = 82% in the de Zeeuw et al. (2016) and 66 + 16 = 82% in the Schwabe et al. (2016) study.

In future research, a method should be developed that makes it possible to estimate also variance due to common-environmental influences. This might for example be possible by adding a random effect for every household in the data set, functioning as a proxy measure for the shared environment. Note that this is not an easy feat in an age where many parents split up, children have two mothers or two fathers (or even three parents), and children grow up in multiple households in different combinations of siblings, half-siblings and unrelated children. Here instead we have tried to capture the most important environmental factors available in the census data that might explain correlation in family members: the cultural and educational background of the parents, the denomination of the school, and the number of children in the same classroom.

So irrespective of study design, twins or census, we find a high heritability for educational achievement in the Netherlands. The high heritability might be explained by the homogeneity in educational opportunities: most schools are tied to a standard curriculum and funded by the government (Sturm et al., 1998; de Zeeuw et al., 2015), which might restrict the variation in school environments, leading to smaller individual differences between (see e.g., Heath et al., 1985; Kovas et al., 2013; Shakeshaft et al., 2013; Colodro-Conde et al., 2015 for a similar argument).

## Author contributions

SvdB developed the study concept and performed the statistical analyses. IS drafted the manuscript and SvdB and LJ provided critical revisions and feedback. All authors approved the final version of the manuscript for submission.

### Conflict of interest statement

The authors declare that the research was conducted in the absence of any commercial or financial relationships that could be construed as a potential conflict of interest.
